# Survey Sequencing and Comparative Analysis of the Elephant Shark *(Callorhinchus milii)* Genome

**DOI:** 10.1371/journal.pbio.0050101

**Published:** 2007-04-03

**Authors:** Byrappa Venkatesh, Ewen F Kirkness, Yong-Hwee Loh, Aaron L Halpern, Alison P Lee, Justin Johnson, Nidhi Dandona, Lakshmi D Viswanathan, Alice Tay, J. Craig Venter, Robert L Strausberg, Sydney Brenner

**Affiliations:** 1 Institute of Molecular and Cell Biology, Singapore; 2 The Institute for Genomic Research, Rockville, Maryland, United States of America; 3 J. Craig Venter Institute, Rockville, Maryland, United States of America; University of California Berkeley, United States of America

## Abstract

Owing to their phylogenetic position, cartilaginous fishes (sharks, rays, skates, and chimaeras) provide a critical reference for our understanding of vertebrate genome evolution. The relatively small genome of the elephant shark, *Callorhinchus milii,* a chimaera, makes it an attractive model cartilaginous fish genome for whole-genome sequencing and comparative analysis. Here, the authors describe survey sequencing (1.4× coverage) and comparative analysis of the elephant shark genome, one of the first cartilaginous fish genomes to be sequenced to this depth. Repetitive sequences, represented mainly by a novel family of short interspersed element–like and long interspersed element–like sequences, account for about 28% of the elephant shark genome. Fragments of approximately 15,000 elephant shark genes reveal specific examples of genes that have been lost differentially during the evolution of tetrapod and teleost fish lineages. Interestingly, the degree of conserved synteny and conserved sequences between the human and elephant shark genomes are higher than that between human and teleost fish genomes. Elephant shark contains putative four Hox clusters indicating that, unlike teleost fish genomes, the elephant shark genome has not experienced an additional whole-genome duplication. These findings underscore the importance of the elephant shark as a critical reference vertebrate genome for comparative analysis of the human and other vertebrate genomes. This study also demonstrates that a survey-sequencing approach can be applied productively for comparative analysis of distantly related vertebrate genomes.

## Author Summary

Cartilaginous fishes (sharks, rays, skates, and chimaeras) are the phylogenetically oldest group of living jawed vertebrates. They are also an important outgroup for understanding the evolution of bony vertebrates such as human and teleost fishes. We performed survey sequencing (1.4× coverage) of a chimaera, the elephant shark *(Callorhinchus milii).* The elephant shark genome, estimated to be about 910 Mb long, comprises about 28% repetitive elements. Comparative analysis of approximately 15,000 elephant shark gene fragments revealed examples of several ancient genes that have been lost differentially during the evolution of human and teleost fish lineages. Interestingly, the human and elephant shark genomes exhibit a higher degree of synteny and sequence conservation than human and teleost fish (zebrafish and fugu) genomes, even though humans are more closely related to teleost fishes than to the elephant shark. Unlike teleost fish genomes, the elephant shark genome does not seem to have experienced an additional round of whole-genome duplication. These findings underscore the importance of the elephant shark as a useful “model” cartilaginous fish genome for understanding vertebrate genome evolution.

## Abstract 3

The cartilaginous elephant shark has a basal phylogenetic position useful for understanding jawed vertebrate evolution. Survey sequencing of its genome identified four Hox clusters, suggesting that, unlike for teleost fishes, no additional whole-genome duplication has occurred.

## Introduction

Our understanding of the human genome has benefited greatly from comparative studies with other vertebrate genomes. Comparison with closely related genomes can identify divergent sequences that may underlie unique phenotypes of human (e.g., [1,2]), while comparison with distantly related genomes can highlight conserved elements that likely play fundamental roles in vertebrate development and physiology. Among the vertebrate taxa that are most distant from human, teleost fishes that shared a common ancestor with tetrapods about 416 million years (My) ago [3,4] have been valuable for discovering novel genes and conserved gene regulatory regions. Several hundred novel human genes were discovered by comparing the human genome with compact genomes of the pufferfishes, fugu and *Tetraodon* [5,6]. Genome-wide comparisons of human–fugu and human–zebrafish have been effective in identifying a large number of evolutionarily conserved putative regulatory elements in the human genome [7,8]. However, comparisons of the human and teleost fish genomes are complicated by the presence of many “fish-specific” duplicate gene loci in teleosts. These duplicate loci have been attributed to a “fish-specific” whole-genome duplication event that occurred in the ray-finned fish lineage approximately 350 My ago [9,10]. The extent and copies of “fish-specific” duplicated genes retained following the fish-specific genome duplication vary in different teleost lineages. For example, genome-wide comparison between zebrafish and *Tetraodon* has shown that different duplicated genes have been retained in these teleosts [11]. Analysis of Hox clusters show that compared to four Hox clusters (HoxA, HoxB, HoxC, and HoxD) with 39 Hox genes in mammals, fugu and zebrafish contain seven Hox clusters with 45 and 49 Hox genes, respectively [12–14]. Fugu has completely lost a copy of the duplicated HoxC cluster, whereas zebrafish has retained both HoxC clusters, and lost a copy of the duplicated HoxD cluster. Adding further complexity, the rates at which specific duplicated genes have mutated vary significantly among different teleost fish lineages [15,16]. Consequently, it is not always straightforward to define orthologous relationships between the genes of teleost fishes and human.

**Figure 1:**
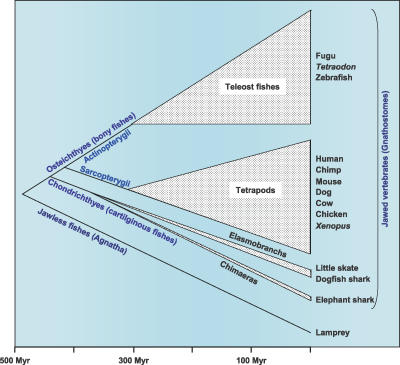
Phylogenetic Tree of Vertebrates The vertical axis represents the abundance of extant species in each of the groups. Names of representative member(s) of each of the lineages are given. The extant Actinopterygii (ray-finned fishes) include Cladistia (e.g., bichir, reedfish), Chondrostei (e.g., sturgeons, paddlefish), Ginglymodi (gars), Amiiformes (bowfin), and Teleostei (e.g., fugu, zebrafish); Sarcopterygii (lobe-finned fishes) include coelacanths, lungfish, and tetrapods (amphibians, birds, reptiles, mammals). Among these, only the teleost and tetrapod branches are shown. The divergence times shown are the minimum divergence times estimated based on fossil records. Agnatha-Gnathostomes, 477 My [69]; Chondrichthyes-Osteichthyes, 450 My [17]; elasmobranchs-chimaeras, 374 My [19]; tetrapods and teleost fishes, 416 My [3,4]. Note that these divergence times are more recent than the molecular sequence–based estimates (e.g., Kumar and Hedges [70]: Agnatha-Gnathostomes, 564 My; Chondrichthyes-Osteichthyes, 528 My; tetrapods and teleost fishes, 450 My).

The living jawed vertebrates (Gnathostomes) are represented by two lineages: the bony fishes (Osteichthyes) and cartilaginous fishes (Chondrichthyes). The bony fishes are divided into two groups, the lobe-finned fishes represented by lungfishes, coelacanths, and tetrapods, and the ray-finned fishes (e.g., teleosts; see Figure 1). The cartilaginous fishes possess a body plan and complex physiological systems such as an adaptive immune system, pressurized circulatory system, and central nervous system that are similar to bony fishes, but distinct from the jawless vertebrates (Agnatha). The oldest fossil record of scales from cartilaginous fishes is dated to be about 450 My old [17]. The living cartilaginous fishes are a monophyletic group comprising two lineages: the elasmobranchs represented by sharks, rays, and skates; and the holocephalians, represented by chimaeras [18]. The two lineages of cartilaginous fishes diverged about 374 My ago [19]. By virtue of their phylogenetic position, cartilaginous fishes are an important group for our understanding of the origins of complex developmental and physiological systems of jawed vertebrates. They also serve as a critical outgroup in comparisons of tetrapods and teleost fishes, and help in identifying specialized genomic features (polarizing character states) that have contributed to the divergent evolution of tetrapod and teleost fish genomes.

A major impediment to the characterization of genomes from cartilaginous fish is their large size. The dogfish shark *(Squalus acanthias),* nurse shark *(Ginglystoma cirratum),* horn shark *(Heterodontus francisi),* and little skate *(Raja erinacea),* which are all popular subjects for biological research, have genome sizes that range from 3,500 Mb to 7,000 Mb [20]. In order to identify a model cartilaginous fish genome that could be sequenced economically, we recently surveyed the genome sizes of many cartilaginous fishes, and showed that the genome of the elephant shark, Callorhinchus milii (also known as the elephant fish or ghost shark) is small relative to other cartilaginous fishes [21]. The elephant shark is a chimaerid holocephalian (Order Chimaeriformes; Family Callorhynchidae) [18]. Their natural habitat lies within the continental shelves of southern Australia and New Zealand at depths of 200 to 500 m. Elephant sharks grow to a maximum length of 120 cm. Mature adults migrate into large estuaries and inshore bays for spawning during spring and summer [22].

To further explore the elephant shark genome, and to evaluate its utility as a model for better understanding the human and other vertebrate genomes, we have conducted survey sequencing and analysis of the elephant shark genome. Previously, a survey sequencing approach was used to estimate several global parameters of the dog genome [23]. Here, we demonstrate that the survey-sequencing approach can also be applied productively for comparative analysis of much more distantly related vertebrate genomes.

## Results

### Sequencing and Sequence Assembly

Whole-genome shotgun sequences for the elephant shark were derived mainly from paired end-reads of 0.85 million fosmid clones. The reads were assembled with the Celera Assembler, yielding 0.33 million contigs and 0.24 million singletons. Contigs that were linked by at least two mated end-reads were ordered within larger scaffolds. The combined length of the assembly, including singletons, is 793.4 Mb. Previously, we estimated the length of euchromatic DNA in the dog genome after survey-sequence coverage (2.43 Gb after 1.5× coverage [23]), and this value is very close to that estimated after more complete sequencing (2.44 Gb after 7.5× coverage [24]). A similar approach (see Materials and Methods) was used to estimate the length of euchromatic DNA in the elephant shark genome (0.91 Gb). This value is similar to the length of the chicken genome (0.96–1.05 Gb [25]), and is consistent with FACScan data that showed elephant shark and chicken genomes are of similar length [21]. Assuming a haploid genome size of 0.91 Gb, the sequence data represents 1.4× coverage, and the assembly output (0.329 million contigs of mean length 1.72 kb) is comparable to a simple model assembly [26] with 40 base overlaps (0.327 million contigs of mean length 1.87 kb). Assuming 1.4× coverage, the maximal possible genome coverage is ∼75%.

### Repetitive Elements

**Figure 2:**
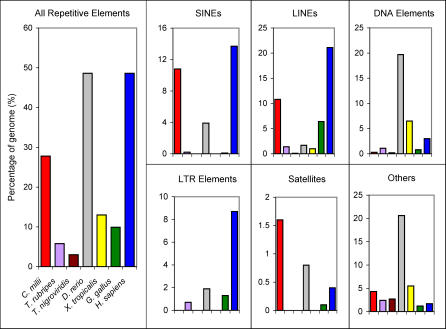
Classification of Repetitive Elements from Several Sequenced Vertebrate Genomes Using RepeatMasker Values (% sequenced genome) for each class of repeats were obtained for Takifugu rubripes (fr1), Tetraodon nigroviridis (tetNig1), *Danio rerio* (danRer3), Xenopus tropicalis (xenTro1), Gallus gallus (galGal2), and Homo sapiens (hg17) from the University of California at Santa Cruz Genome Bioinformatics Site (http://www.genome.ucsc.edu). Callorhinchus milii repeats were identified in the 1.4× sequence generated in this study.

RepeatMasker (version 3.0.8; http://www.repeatmasker.org) uses a library that includes 310 known repeats from Chondrichthyes and Actinopterygii (ray-finned fishes). However, the elephant shark genome contains few homologs of these characterized repeats, and only 6.0% of the elephant shark sequence was classified by RepeatMasker as repetitive (including 3.0% that is merely simple or low-complexity sequence). In order to estimate the content of novel repetitive elements, a sample of 100,000 sequence reads was searched against itself using BLASTN. Reads that matched more than 500 other reads were aligned to build consensus sequences for novel repetitive elements. This yielded ten unique consensus sequences, consisting of two short interspersed element (SINE)–like repeats, three long interspersed element (LINE)–like repeats, four satellite-like sequences, and one sequence of unknown identity. When these ten sequences were added to the 310 known fish repeats, RepeatMasker classified 27.8% of the elephant shark assembly as repetitive. Among the genomes of vertebrates, the content of retrotransposons in elephant shark appears to be much higher than for other nonmammalian species (Figure 2). However, these values are dependent on the level of curation that has been applied to the repeats of each genome, which may not be uniform. The most abundant SINE and LINE-like species each have homology with 7%–8% of the elephant shark genome. The SINE appears to be tRNA-derived, while the LINE encodes a reverse transcriptase with greatest similarity to CR1-like retrotransposons from fish [27]. Like several other vertebrate species [28], the major SINE and LINE species of elephant shark share significant sequence homology at their 3′ ends (41 of 46 identical bases).

### Protein-Coding Genes

The content of protein-coding genes was assessed by comparing the translated assembly with known and predicted protein sequences. Nonrepetitive sequences were searched against annotated proteins from the genomes of human, chicken, fugu, zebrafish, *Ciona intestinalis,* fruit fly, and nematode, and all known proteins from cartilaginous fishes. A total of 60,705 “genic regions” were identified, with a majority representing partial gene sequences. Of the 608,147 sequences in the assembly, 55,298 contain a single genic region each, and 2,663 contain two or more genic regions. The combined length of coding sequence in these genic regions is 20.6 Mb, representing 2.6% of the assembled sequence data. This value is likely an underestimate because the homology-based approach used would fail to identify genes that are evolving faster than their homologs in other genomes. For example, when a homology-based approach was used to annotate the fugu genome, it failed to identify homologs for nearly 25% of human genes, particularly the cytokine genes, in the fugu genome [5]. However, many of these genes were subsequently identified in another pufferfish *(Tetraodon)* based on sequencing of cDNAs [6]. We therefore expect the fraction of coding sequences in the elephant shark genome to be greater than 2.6%.

We assigned putative orthology to genic regions based on their best matching protein sequences in other genomes. However, different fragments of the same gene can display best matches to proteins from different genomes. To avoid this redundancy, we first searched the conceptual protein sequences against the nonredundant human proteome. Of the 60,705 genic regions, 48,400 (80%) had significant similarity (cutoff at 1 × 10^−10^) to 11,805 human proteins. For the remaining genic regions, the assignment of putative orthology was based on significant matches to known proteins in cartilaginous fishes, chicken, fugu, zebrafish, and *C. intestinalis.* In total, the genic regions of the elephant shark assembly contain partial or complete sequences for 14,828 genes. This collection defines a minimal set of elephant shark genes that share strong sequence similarity with known vertebrate genes. A description of these genes can be found at http://esharkgenome.imcb.a-star.edu.sg.

Annotation of InterPro domains within the putative protein sequences identified 3,085 unique domains (http://esharkgenome.imcb.a-star.edu.sg). Most of these domains are also found in annotated proteins of human, mouse, dog, fugu, *Tetraodon,* and zebrafish. However, 26 domains are absent only from teleost fishes (Table S1), five domains are absent only from mammals (Table S2), and ten domains are absent from both teleost fishes and mammals (Table S3). The elephant shark protein domains absent from teleost fishes or mammals are likely to be encoded by genes that have been lost, or have diverged extensively, in these lineages.

### Elephant Shark and Human Genes Lacking Orthologs in Teleost Fishes

Cartilaginous fishes are a useful outgroup for comparison of tetrapod and teleost fish genomes (Figure 1). Comparisons of the gene complements for elephant shark, mammals, and teleost fishes should help to identify ancient genes shared by the three groups of jawed vertebrates and genes that have undergone differential loss or expansion in mammalian and teleost fish lineages. Our analysis (see Materials and Methods) identified 154 human genes that have orthologs in mouse, dog, and the elephant shark, but not in the teleost fish genomes (Table S4). Out of the 154 genes, 85 (highlighted in Table S4) have no homologs in *C. intestinalis,* fruit fly, or the nematode worm. These are likely to be vertebrate-specific genes that have been lost (or are highly divergent) in the teleost lineage. Among these genes are notable examples, such as ribonuclease L *(RNaseL)* and 2′-5′oligoadenylate synthetase 1 *(2′-5′OAS).* The enzymes encoded by these genes are thought to play an important role in the innate immune response to viral infection. 2′-5′OAS is induced by interferon, and activated by double-stranded RNA [29]. Its activity catalyzes the synthesis of oligoadenylates that activate the latent endoribonuclease, RNaseL. The activated RNase degrades both viral and cellular RNA, and is thought to mediate apoptosis. Previously, the genes encoding 2′-5′OAS and RNaseL had been identified only in mammals and chicken. Orthologs of the two enzymes were not identified in the genomes of the three sequenced teleost fishes, or the amphibian, Xenopus tropicalis (http://www.ensembl.org). This suggests that the relevant genes have been lost independently from at least two vertebrate lineages.

This set of genes also includes three members of the amiloride-sensitive epithelial Na^+^ channel (ENaC) family. This family includes four members, ENaC α , β, γ, and δ subunits, and all members have been cloned from mammals, birds, and amphibians. However, none has been identified in teleost fishes. In contrast to the voltage-gated sodium channels that generate electrical signals in excitable cells, ENaC channels mediate electrogenic transport of Na^+^ across the apical membranes of polarized epithelial cells. The active transepithelial transport of Na^+^ is important for maintaining Na^+^ and K^+^ levels in the kidney and colon [30]. The mechanism of Na^+^ uptake in teleost fish cells is currently a subject of controversy. Two models have been proposed. The original model involves amiloride-sensitive electroneutral Na^+^/H^+^ exchanger (NHE), with the driving force derived from Na^+^-K^+^ ATPase and carbonic anhydrase [31]. A recent model involves ENaC, electrochemically coupled to H^+^-ATPase [32]. This is not supported by our observation of the loss of ancestral ENaC subunit genes from teleost fish genomes. On the other hand, since *NHE* has been cloned from a teleost fish, and is shown to express at high levels on the apical membrane of chloride cells [33], the original model seems to be a likely mechanism for Na^+^ uptake in teleost fishes.

A significant number of human genes that have orthologs in the elephant shark but not in teleost fishes are associated with male germ cells and fertilization (Table 1). These include genes that encode zona pellucida (ZP)–binding protein (Sp38) and ZP–sperm-binding protein (ZP-1). These are respectively expressed in the acrosome of sperm [34] and the ZP of oocytes [35] where they mediate the binding of sperm to ZP. In mammals, several sperm initially bind to ZP but only one of them triggers the “acrosomal reaction” that leads to successful fertilization and prevention of other sperm from entering the oocyte. In contrast, sperm of teleost fishes enter the egg through a unique structure called the micropyle, which allows only one sperm to enter and fertilize the oocyte [36]. Micropyle does not exist in the oocytes of mammals and cartilaginous fishes. The conservation of genes essential for the binding of sperm to ZP in mammals and the elephant shark indicates that cartilaginous fishes use the ZP-mediated mode of fertilization similar to mammals. These genes seem to have been either lost or become divergent in teleost fishes following the invention of the micropyle.

**Table 1:**
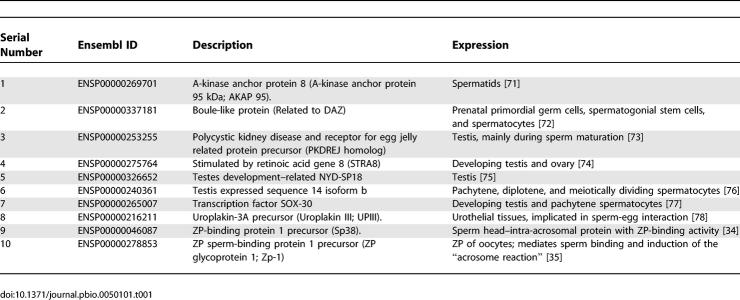
Human Proteins Known to Be Associated with Male Germ Cells, and Present in the Elephant Shark but Divergent or Absent in Teleost Fishes

### Elephant Shark and Teleost Fish Genes Lacking Orthologs in Mammals

Our analysis identified 107 teleost fish genes that have orthologs in the elephant shark assembly, but not in the human, mouse, and dog genomes (Table S5). Twenty of these genes have no homologs in invertebrate genomes (*C. intestinalis,* fruit fly, and nematode worm) and are likely to be vertebrate-specific. The remaining 87 genes (Table S5) are ancient metazoan genes that have been conserved in the elephant shark and teleost fishes, but were lost or are highly divergent in the mammalian lineage. The loss of the ancient vertebrate-specific genes in mammals is likely to be related to some of the divergent phenotypes of mammals compared with cartilaginous fishes and teleost fishes. The vertebrate-specific genes absent from mammals include *globinX (GbX),* the recently identified fifth member of the vertebrate globin family that includes hemoglobin, myoglobin, neuroglobin, and cytoglobin. *GbX* has been cloned from teleost fishes and amphibians but has been reported to be absent in amniotes [37]. Although *GbX* shows expression in several nonneuronal tissues, its function is unknown. The existence of *GbX* in the elephant shark has confirmed that this is an ancient vertebrate gene that has been lost from the amniote lineage. The genes that are absent from mammals include a large number (80 of 107) that are either hypothetical or predicted novel genes with no known function (Table S5). It is possible that some of these genes may be necessary for aquatic life and should be targeted for functional analysis.

### Conserved Synteny

After 1–2× sequence coverage of vertebrate genomes using conventional plasmid clones, the assembled sequence data has little long-range continuity that can be used to identify conserved synteny between species. For example, 1.5× coverage of the dog genome yielded scaffolds with a mean span of only 8.6 kb [23]. For our survey of the elephant shark genome, >95% of the sequence data was derived from fosmid clones, with inserts of 35–40 kb. Consequently, it was possible to derive much more information on the relative ordering of sequenced genes. For 10,708 fosmid clones, the paired end-reads are located in contigs that have significant homology to unique pairs of human genes. For most pairs (10,655), both genes have defined chromosomal locations. These include 3,059 unique pairs of genes (29%) that are separated by less than 1 Mb on the human genome (median separation, 48 kb). These 3,059 gene pairs could be collapsed further into 1,713 clusters, containing a total of 4,629 genes, in clusters of two to 23 genes per cluster (http://esharkgenome.imcb.a-star.edu.sg). For comparison, conserved synteny between the elephant shark and zebrafish genomes was analyzed. There was a similar number of fosmid clones (13,773) with end-reads in contigs that have significant homology to unique pairs of zebrafish genes. For 7,916 pairs, both genes have defined chromosomal coordinates. Interestingly, only 848 of these gene pairs (11%) are separated by <1 Mb in the zebrafish genome (median separation, 22 kb), and these are consolidated into 657 clusters, containing 1,489 genes in clusters of two to six genes per cluster (http://esharkgenome.imcb.a-star.edu.sg). When normalized to the number of unique gene pairs with defined chromosomal coordinates, the level of detectable conserved synteny for human is more than double that seen for zebrafish. These data suggest that elephant shark genome has experienced a lower level of rearrangements compared to teleost fish genomes. This is consistent with the observation that the major histocompatibility complex (MHC) class I and class II genes that are closely linked in mammals and cartilaginous fish such as nurse shark and banded houndshark *(Triakis scyllium)* are located on different chromosomes in zebrafish, carp, trout, and salmon [38]. Loss of some syntenic blocks in teleost fish could be explained by the differential loss of duplicate genes that arose due to a “fish-specific” whole-genome duplication event in the ray-finned fish lineage [9,10]. For instance, conserved synteny of genes X-Y between the elephant shark and human genomes could be lost in teleost fishes if alternative copies of duplicate genes on paralogous chromosome segments containing duplicate Xa-Ya and Xb-Yb genes are lost resulting in Xa-† and †-Yb genes († represents the lost gene). The higher level of synteny conservation between the elephant shark and human suggests that the elephant shark genome has not undergone whole-genome duplication, and that the identification of orthologous genes in the genomes of elephant shark and nonteleost vertebrates will benefit from the analysis of conserved synteny.

### UCEs in the Elephant Shark Genome

Bejerano et al. [39] have identified 481 ultraconserved elements (UCEs) that are longer than 200 bp and perfectly conserved among the human, mouse, and rat genomes. These UCEs overlap transcribed and nontranscribed regions of the genome. To assess the extent of UCEs conserved in the cartilaginous fish genomes, we searched for UCEs in the elephant shark sequences, and fugu and zebrafish genomes (see Material and Methods). Of the 481 UCEs, 57% are found in the elephant shark sequences (83% coverage with an average identity of 86%), whereas 55% and 62% are found (81% coverage, average identity 84%) in the fugu and zebrafish, respectively. Of the 141 UCEs missing from both fugu and zebrafish, 46 (33%) are found in the elephant shark sequences. We predict that the whole genome of the elephant shark will contain ∼75% of the UCEs. Our analysis of the noncoding sequences in the elephant shark has shown that the elephant shark and human genomes contain twice as many conserved noncoding elements as that between human and zebrafish or fugu [40]. Taken together, these results suggest that a higher proportion of human sequences might be conserved in the elephant shark genome than in the teleost fish genomes.

### Adaptive Immune System Genes

**Figure 3:**
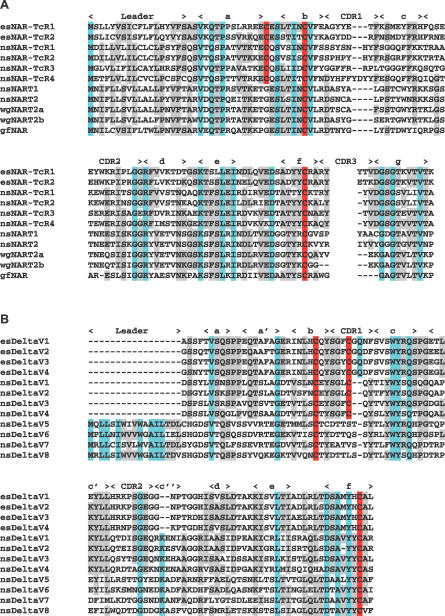
NAR-TcR Genes in the Elephant Shark (A) Alignment of predicted amino acid sequences of some representative elephant shark NAR-TcRV (esNAR-TcR1 and esNAR-TcR2) with their homologs from the nurse shark (nsNAR-TcR1 to nsNAR-TcR4) and IgNARV sequences from nurse shark (nsNART1 and nsNART2), wobbeygong shark (wgNART2a and wgNART2b) and guitarfish (gfNAR). Alignment of CDR3s, which are highly variable in sequence and length, is not shown. (B) Alignment of predicted amino acid sequences of putative elephant shark NAR-TcRV–associated TcRδV (esDeltaV1 to esDeltaV4) with nurse shark NAR-TcRV–associated TcRδV sequences (nsDeltaV1 to nsDeltaV4), and typical nurse shark TcRδV sequences (nsDeltaV5 to nsDeltaV8). Leader regions, β-strands, and complementarity-determining regions (CDRs) are indicated above each alignment. Conserved residues are highlighted in blue and gray, and conserved cysteine residues in immunoglobulin superfamily canonical intradomain and putative interdomain disulfide bridges are highlighted in red. Note the conserved cysteine residue in the a-b loop and the absence of the canonical tryptophan of the “WYRK” motif in the NAR-TcRV sequences (alignment A). The NAR-TcRV–associated TcRδV lacks the leader peptide, and encodes a conserved cysteine residue in the CDR1 (alignment B). Sequences of nurse shark, wobbeygong shark, and guitarfish are taken from Criscitiello et al. [43].

Cartilaginous fishes are the phylogenetically oldest group of living organisms known to possess an adaptive immune system based on rearranging antigen receptors. They possess all the four types of T-cell receptors identified in mammals (TcRα, β, γ, and δ); at least three types of Ig isotypes: IgM, IgW (also called IgX-long or IgNARC in some species) and new antigen receptor (IgNAR); the recombination-activating genes *(RAG1* and *RAG2)*; and polymorphic MHC genes. The IgNAR isotype, found only in cartilaginous fishes, is unique in that it does not form a heterotetramer (of two light chains and two heavy chains) but instead forms a homodimer of two heavy chains and binds to antigen as a single V domain [41]. A major difference between cartilaginous fishes and other jawed vertebrates is in the organization of Ig genes. In other jawed vertebrates each Ig locus is organized as a single “translocon” containing all the V genes in the 5′ region, followed by all the D, J, and then C region genes in the 3′ end. In contrast, the Ig genes in cartilaginous fishes are present in multiple “clusters,” with each cluster typically consisting of one V, two D, one J, and one set of C exons [42]. In addition to the above distinct types of Ig and TcR antigen receptor chains, a unique antigen receptor chain comprising two V domains called new antigen receptor–T-cell receptor V domain (NAR-TcRV) and TcRδ V domain (TcRδV) has been recently identified in the nurse shark [43]. The two V domains in the NAR-TcR chain contain a combination of characteristics of both IgNAR and TcR and are generated by separate VDJ gene rearrangements. Such a combination between the Ig and TcR antigen receptor chains were previously thought to be incompatible. BLAST searches of the elephant shark assembly showed that the elephant shark contains homologs for all known cartilaginous fish adaptive immune system genes except *IgNAR* (see descriptions of genes at http://esharkgenome.imcb.a-star.edu.sg). Since the elephant shark genome sequence is incomplete, it is unclear whether *IgNAR* genes are absent in the elephant shark. The discovery of the *NAR-TcR* genes in the elephant shark assembly is particularly significant since previous attempts to identify this gene in the spotted ratfish *(Hydrolagus colliei),* a chimaera, by Southern blot analysis using probes from the nurse shark had suggested that this family may be absent in chimaeras [43]. Alignments of peptide sequences of representative elephant shark NAR-TcRVs and associated TcRδVs, together with their homologs from the nurse shark, are shown in Figure 3. Similar to the nurse shark NAR-TcRV, the peptides encoded by the elephant shark *NAR-TcR* gene contain a typical leader peptide and a cysteine residue in the a-b loop, and lack the canonical tryptophan of the “WYRK” motif. The associated elephant shark TcRδVs lack the leader peptide and share a conserved cysteine residue in the CDR1 similar to their nurse shark homologs (Figure 3). The identification of homologs of *NAR-TcR* in the elephant shark confirms that this unique doubly rearranging antigen receptor evolved in a common ancestor of elasmobranchs and chimaeras.

### Hox Genes in the Elephant Shark Genome

**Figure 4:**
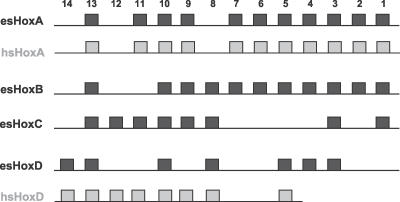
Hox Genes in the Elephant Shark and Horn Shark Hox genes belonging to putative four Hox clusters (esHoxA to esHoxD) identified in the elephant shark assembly are shown as dark filled boxes. Linkage of genes is inferred from linkage information in horn shark and other vertebrates. For comparison, horn shark HoxA and HoxD cluster (hsHoxA and hsHoxD) genes (light filled boxes) are shown [51,52]. In the horn shark, only a partial HoxD cluster (from *HoxD5* to *HoxD14*) has been sequenced, and HoxB and HoxC clusters are yet to be sequenced.

Hox genes are transcription factors that play a crucial role in the control of pattern formation along the anterior–posterior axis of metazoans. In vertebrates and most nonvertebrates, Hox genes are arranged in clusters and thus are central to the characterization of genome duplications during vertebrate evolution. The amphioxus, a cephalochordate, contains a single cluster of 14 Hox genes [44], whereas coelacanth (a lobe-finned fish) and mammals contain four Hox clusters (HoxA, HoxB, HoxC, and HoxD) that have arisen through two rounds of duplication during the evolution of vertebrates [45,46]. Teleost fishes such as zebrafish and pufferfish contain almost twice the number of Hox clusters found in mammals [5,6,47], due to the additional “fish-specific” whole-genome duplication in the ray-finned fish lineage [9,10]. Jawless vertebrates (e.g., the sea lamprey) contain at least three Hox clusters [48,49], one of which seems to be the result of a lineage-specific duplication event [50]. Among the cartilaginous fishes, a complete HoxA cluster and a partial HoxD cluster *(HoxD5* to *HoxD14)* have been sequenced from the horn shark [51,52]. The total number of Hox clusters and Hox genes in cartilaginous fishes is currently unknown. Hox genes typically consist of two exons, and their orthology can be identified reliably based even only on the second exon, which codes for the Hox domain. We identified Hox genes in the elephant shark assembly using a combination of manual annotation, reciprocal BLAST searches, and phylogenetic analysis. A total of 37 partial or complete sequences of Hox genes that were located on different contigs could be identified. These genes belong to putative four Hox clusters (HoxA, HoxB, HoxC, and HoxD), and include a maximum of four members for each of the 14 paralogy groups (Hox1 to Hox14; Figure 4). Thus, elephant shark is likely to contain only four Hox clusters similar to coelacanth and mammals. The presence of four Hox clusters in the elephant shark suggests that, unlike teleost fishes, the elephant shark lineage has not experienced additional whole-genome duplication.

**Figure 5:**
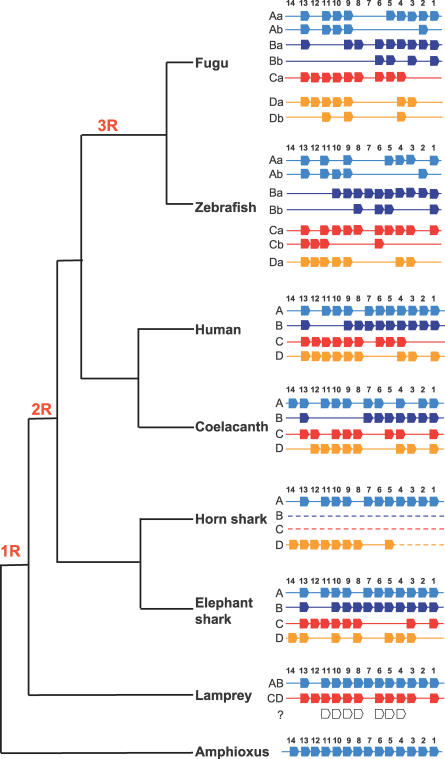
The Evolution of Vertebrate Hox Cluster Organization and the History of Whole-Genome Duplications in Vertebrates The additional Hox genes in the lamprey (shown as white arrows) are the result of a lineage-specific duplication of the “AB” or “CD” Hox cluster. In the horn shark, only a partial HoxD cluster (from *HoxD5* to *HoxD14*) has been sequenced, and HoxB and HoxC clusters are yet to be sequenced. The number of Hox clusters in various lineages is consistent with one round of genome duplication (“1R”) during the evolution of jawless vertebrates from chordate invertebrates, a second round (“2R”) before the emergence of jawed vertebrates but after the divergence of the lamprey lineage, and a third round (“3R”) in the ray-finned fish lineage before the divergence of zebrafish and fugu lineages. See text for the source of Hox cluster information.

Although Hox genes identified in the elephant shark assembly may not include all the Hox genes in the genome, they provide the first glimpse of Hox genes belonging to the four clusters in a cartilaginous fish. The HoxA cluster genes identified in the elephant shark include orthologs of all the HoxA genes identified in the horn shark, while the elephant shark HoxD cluster genes include two genes *(HoxD3* and *HoxD4)* whose orthologs are yet to be identified in the horn shark (Figure 4). The elephant shark HoxB and HoxC cluster genes are the first members of these clusters to be identified in a cartilaginous fish. Comparisons of the elephant shark Hox genes with genes from the completely sequenced Hox clusters from mammals and ray-finned fishes have identified several Hox genes that have been differentially lost in mammals and ray-finned fishes (Figure 5). For example, *HoxD5* and *HoxD14* genes present in the elephant shark have been lost in both mammalian and teleost lineages, whereas *HoxA6*, *HoxA7,* and *HoxD8* have been lost only in the teleost lineage. Interestingly, the single HoxA cluster in a nonteleost ray-finned fish, bichir, contains a functional *HoxA6* gene and a *HoxA7* pseudogene, indicating that *HoxA6* was lost in the ray-finned fish lineage after the divergence of the bichir lineage [53]. An ortholog for the elephant shark *HoxC1* gene is absent in both mammals and fugu, and is on the way to becoming a pseudogene in zebrafish [54]. However, the presence of this gene in the coelacanth indicates that it has been lost independently in the mammalian lineage after the divergence of the coelacanth and in the lineage leading to teleosts. The presence of *HoxB10* in the elephant shark and zebrafish and its absence in mammals and fugu suggest that this gene was lost independently in the teleost lineage leading to fugu after the divergence of the zebrafish lineage and in the mammalian lineage. These comparisons show that duplication of Hox clusters and differential loss of Hox genes is a continuous process in the evolution of vertebrates. The ancestral jawed vertebrate Hox genes that have been differentially lost in different lineages are potential targets for studies aimed at understanding the molecular basis of morphological phenotypic differences between different vertebrate lineages.

## Discussion

The extant jawed vertebrates are represented by three major lineages, the cartilaginous fishes, the lobe-finned fishes, and the ray-finned fishes, with the cartilaginous fishes constituting an outgroup to the other two groups. Cartilaginous fishes thus constitute a critical reference for understanding the evolution of jawed vertebrates. The survey sequencing of the elephant shark, the first cartilaginous fish genome to be characterized to this depth, has provided useful information regarding the length, gene complement, and organization of the genome, and highlighted specific examples of vertebrate genes and gene families that have been lost differentially in the mammalian and teleost fish lineages. The 1.4× coverage elephant shark sequence generated in this study contains partial or complete sequences for about 15,000 unique genes. These sequences can serve as probes for isolating genomic clones and for obtaining complete sequences of gene loci of interest on a priority basis. At 0.91 Gb, the length of elephant shark genome is similar to that of the chicken (1.05 Gb), half that of the zebrafish (∼1.7 Gb), and one-third the length of the human genome (2.9 Gb). It is about twice the length of the fugu and *Tetraodon* genomes (∼0.4 Gb), which are the smallest among vertebrates. The elephant shark genome is the smallest among known cartilaginous fish genomes, and thus is an ideal cartilaginous fish genome for economically sequencing the whole genome and for comparative analysis.

A major drawback in comparisons between human and teleost fish genomes is the presence of many duplicate gene loci in teleost fishes due to the additional fish-specific whole-genome duplication event in the ray-finned fish lineage. Analysis of Hox genes in the elephant shark assembly has indicated that the elephant shark genome has not undergone a lineage-specific whole-genome duplication. Interestingly, the human and elephant shark genomes exhibit a higher level of conserved synteny compared with human and zebrafish genomes, even though humans are more closely related to zebrafish than they are to the elephant shark. The disruption of syntenic blocks in the teleosts may be partly related to differential loss of duplicate copies of genes following the fish-specific genome duplication event. The elephant shark also exhibits a higher level of sequence similarity with humans. A higher number of mammalian UCEs, which include both coding and noncoding sequences, were identified in the elephant shark genome compared with the zebrafish and fugu genomes. In a related study, we have shown that twice as many noncoding elements are conserved between human and elephant shark genomes compared with that between human and zebrafish or fugu genomes [40]. The higher level of sequence similarity between the elephant shark and humans could be due to a decelerated evolutionary rate of the elephant shark DNA compared with human and teleost DNA or an accelerated evolutionary rate of teleost sequences compared with the elephant shark and human genomes. Analysis of mitochondrial DNA sequences from 12 lineages of sharks belonging to the elasmobranch lineage has shown that the nucleotide substitution rate in sharks is 7- to 8-fold slower than in mammals [55]. The evolutionary rate of mitochondrial proteins ND2 and Cytb was also found to be slower (about one-fourth) in these sharks compared with mammals [56]. These studies suggest that the evolutionary rate of DNA in cartilaginous fishes is slower than that in mammals. Comparisons of evolutionary rates of protein-coding genes in *Tetraodon,* fugu, zebrafish, and other teleosts have shown that the fish coding sequences have been evolving at a faster rate than their mammalian orthologs, and that the duplicated pairs of fish genes are evolving at an asymmetric rate [6,15,16,57,58]. Duplicated fish genes also tend to accumulate complementary degenerate mutations in the coding and noncoding sequences, resulting in partitioning of regulatory elements and exons between the two copies [59–62]. Such partitioning could result in a reduced level of sequence conservation between each of the duplicate copies and its ortholog in humans. Thus, the higher level of sequence similarity between the elephant shark and humans compared with that between teleost fish and humans could be the result of both a decelerated evolutionary rate of elephant shark DNA and an accelerated evolutionary rate of teleost fish sequences. The higher degree of conservation of synteny and conserved sequences between the human and elephant shark genomes compared with human and teleost fish genomes, and the absence of evidence for a lineage-specific whole-genome duplication event in the elephant shark lineage, underscore the importance of the elephant shark genome as a model jawed vertebrate genome for comparative analysis of human and other jawed vertebrate genomes.

Cartilaginous fishes are the oldest phylogenetic group of jawed vertebrates that possess an adaptive immune system. Analysis of the elephant shark genome sequences has identified all components of the adaptive immune system genes (e.g., T-cell receptors, immunoglobulins, and *RAG* and MHC genes) known in tetrapods and teleosts, as well as a unique family of doubly rearranging antigen receptor (NAR-TcR) genes previously reported only in elasmobranch cartilaginous fishes [43]. The presence of this unique family of genes in the elephant shark, a holocephalian, indicates that NAR-TcR existed in a common ancestor of all cartilaginous fishes. Thus, cartilaginous fishes appear to have evolved a distinct type of adaptive immune system after they diverged from their common ancestor with bony fishes. The physiological significance of such a unique adaptive immune system remains to be understood.

The number of Hox gene clusters in vertebrates illuminate the history of genome duplications during vertebrate evolution (Figure 5). It has been proposed that the evolution of phenotypic complexity in vertebrates was accomplished through two rounds of whole-genome duplication (the “2R” hypothesis) during the evolution of vertebrates from invertebrates [63]. Although the presence of four mammalian paralogs for many single genes in invertebrates [64] and four Hox clusters in mammals compared with a single Hox cluster in amphioxus is consistent with this hypothesis, the exact timings of the two rounds of genome duplication are unclear. The identification of four putative clusters of Hox genes in the elephant shark in the present study indicates that the two rounds of genome duplication occurred before the divergence of the cartilaginous fish and bony fish lineages (Figure 5). Since the analyses of Hox genes in jawless vertebrates such as the lamprey show that at least one round of genome duplication (“1R”) occurred before the divergence of the jawless and jawed vertebrate lineages, it can be inferred that the second round of duplication (“2R”) occurred after the divergence of the jawless and jawed vertebrate lineages but before the split of cartilaginous fish and bony fish lineages (Figure 5). The presence of almost twice the number of Hox clusters in teleost fishes as in mammals and the elephant shark supports an additional whole-genome duplication event in the ray-finned fish lineage. This more recent fish-specific genome duplication event, referred to as “3R,” has been hypothesized to be responsible for the rapid speciation and diversity of teleosts [61]. Thus, genome duplication has continued to play an important role in the evolution of vertebrates even after the emergence of bony vertebrates.

In this project, we have taken a survey sequencing approach to characterize the elephant shark genome. Previously, a survey sequencing approach was used to estimate several global parameters of the dog genome, such as its length, repeat content, and neutral mutation rate [23]. The coverage (1.5×) included partial sequence data for dog orthologs of ∼75% of annotated human genes, and revealed that >4% of intergenic sequence is conserved between the dog, human and mouse. More complete sequencing of the dog genome has confirmed the accuracy of these estimates [24]. The survey sequencing approach has now been recognized as an effective and economical way of rapidly characterizing the large genomes of closely related vertebrates for which there is little or no genomic sequences or genetic/physical maps. Here, we have shown that a survey sequencing approach can also be productively used for characterizing most distantly related vertebrate genomes. In contrast to sequencing of paired-ends of short-insert plasmid libraries in conventional whole-genome shotgun sequencing strategy, survey sequencing of the elephant shark genome was based on sequencing of paired-end sequences of fosmid clones. This approach allows accurate assembly of dispersed repeats that are larger than 2–3 kb and provides long-range linkage information that can be used to determine conserved synteny between species. Fosmid clones are also valuable templates for filling gaps in the assembly and for obtaining complete sequences of gene loci of interest. We propose survey sequencing to a depth of 1.5–2× based on paired-end sequencing of large-insert libraries as an effective and economical approach for characterizing distantly related vertebrate genomes.

## Materials and Methods

### Sequencing and sequence assembly.

Genomic DNA was extracted from the testis of an adult elephant shark collected in Hobart, Tasmania. Fosmid libraries (containing 35- to 40-kb inserts) and a plasmid library (3- to 4-kb inserts) were prepared from sheared genomic DNA. End sequencing of clones from each library was conducted using standard procedures, and yielded 1.54 million reads (93.7% paired) from the fosmid clones, and 0.20 million reads (93.1% paired) from the plasmid clones. The finished sequence data consisted of 1.73 million reads, with a mean read length of 763 bases. The reads were assembled with Celera Assembler (http://wgs-assembler.sourceforge.net) [23,65,66]. The assembly output consisted of 0.327 million contigs (mean length, 1,720 bases; mean content, 4.3 reads per contig), 0.245 million singletons, and 0.037 million mini-scaffolds (paired end-reads that were otherwise unassembled). A small number of contigs (2,113) that were linked by at least two mated end-reads were ordered within scaffolds that spanned a total of 33.6 Mb.

### Estimation of genome length.

Previously, we estimated the length of euchromatic DNA in the dog genome after survey sequence coverage (2.43 Gb after 1.5× coverage [23]), and this value is very close to that estimated after more complete sequencing (2.44 Gb after 7.5× coverage [24]). A similar approach was used to estimate the length of euchromatic DNA in the elephant shark genome. The numbers and positions of overlaps that began five or more bases downstream from the 5′ end of each of 200,000 reads were computed. In order to eliminate reads from repetitive regions, only “qualifying” reads with fewer than *k =* 5 overlaps beginning in this region were considered. For the first 100 bases of the region, the number of overlaps beginning in that window is tabulated for each of the *N* qualifying reads. Letting *n_i_* equal the number of qualifying reads with *i* overlaps, the mean number of overlaps per read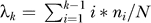 is calculated. For the current dataset, λ_5_ = 0.18 ± 0.01. Although for *k = 5* the effect is small, *λ_k_* is an underestimate due to the truncation of the sum at *i = k −* 1. To correct for this truncation, *λ_k_ = λ_k_′*/*P*(*x < k|λ_k_′*) may be solved for a final estimate, λ*_k_′*. Here, λ*_k_′* = 0.19. Equating *λ_k_′* to *np,* the mean of the binomial distribution, with *n* = 1,730,917 reads, and probability of a read beginning in a window of length 100 being *p* = 100/*G_k_*, where *G_k_* is the estimated genome length, yields *G_k_* = 100*n/λ_k_′* (i.e., *G*_5_ = 9.1 × 10^8^). Estimates based on other values of *k,* ranging from 3 to 6, result in very similar estimates. The assembly output (0.329 million contigs of mean length 1.72 kb) is comparable to a simple model assembly [26] with 40 base overlaps (0.327 million contigs of mean length 1.87 kb).

### Protein-coding genes.

We first delineated “genic regions” in the elephant shark sequences by mapping the extreme start and end positions of individual protein matches from BLASTX alignments. Overlapping genic regions were then clustered to identify the longest non-overlapping genic regions. All the BLASTX high-scoring segment pairs (HSPs) that lay within a genic region were grouped together, and the best matching non-overlapping HSPs were retained to represent the coding regions in that particular genic region. The conceptual protein sequences of HSPs that fall within each genic region were joined to obtain the protein sequences encoded by the genic regions. These genic regions may include some pseudogenes that have retained significant homology to their parent genes. Protein domains in the elephant shark proteins were predicted using the FPrintScan, ScanRegExp, and HMMPfam applications of the InterProScan (version 4.0; http://www.ebi.ac.uk/InterProScan) package. The InterPro domains predicted in human, mouse, dog, fugu, *Tetraodon,* and zebrafish were extracted from Ensembl version 35 (http://www.ensembl.org) and compared with the elephant shark InterPro domains.

### Elephant shark genes lacking orthologs in teleost fishes or mammals.

To identify genes that are orthologous in the elephant shark and mammals, but absent from teleost fishes, we started with 3,708 human genes that have annotated orthologs in the genomes of dog and mouse, but not fugu, *Tetraodon,* or zebrafish (Ensembl, version 35). These genes were used for reciprocal BLAST searches, consisting of a TBLASTN search of the human proteins against the elephant shark assembly (1 × 10^−7^ cutoff), followed by a BLASTX search of the aligned elephant shark sequences against the human proteome (1 × 10^−7^ cutoff). Putative orthologs for 423 of the human genes were found in the elephant shark assembly. In order to discount genes that have partial homologs in fugu, *Tetraodon,* or zebrafish, the 423 human protein sequences were again searched against the three fish genomes using TBLASTN at a less stringent cutoff of 1 × 10^−3^. These assemblies of fugu, *Tetraodon,* and zebrafish genomes are predicted to contain 22,008, 28,005, and 22,877 protein-coding genes, respectively. Of the 423 proteins, 85 had no significant similarity to any of the genomes. The remaining 338 human proteins had similarity to sequences in at least one of the fish genomes. A reciprocal BLASTX search of these fish sequences indicated that 69 of them showed significant similarity to a different sequence in the human proteome. These fish sequences contain domains that are shared by multiple proteins in addition to their true orthologs. To identify genes that are conserved in the elephant shark and teleost fishes, but divergent or lost from mammals, we first identified 2,967 zebrafish genes that have annotated orthologs in the genomes of fugu and *Tetraodon,* but not human, mouse, and dog (Ensembl, version 35). Reciprocal BLAST searches were conducted using the approach described for orthologs that are absent from teleost fishes.

### Conserved synteny.

All elephant shark contigs and singletons (571,269) and miniscaffold reads (73,756 from 36,878 miniscaffolds) were searched against Ensembl-predicted peptides (version 37) from the human genome (National Center for Biotechnology Information version 35; 33,869 peptides from 22,218 genes) and the zebrafish genome (Zv5; 32,143 peptides from 22,877 genes) using BLASTX [67]. Zebrafish was chosen as a representative teleost for this analysis since more genes in the zebrafish assembly have been assigned chromosome coordinates (18,009 of 22,877 predicted) compared to *Tetraodon* (16,275 of 28,005 predicted) and fugu (no chromosome coordinates) assemblies. For the search against human peptides, 122,804 elephant shark sequences produced good alignments with e < 1 × 10^−6^ and a HSP of >50 bits. Of the clones that contributed to these sequences, there were 10,708 where both end reads were linked to unique pairs of human proteins. For the search against zebrafish peptides, 92,291 elephant shark sequences produced good alignments with e < 1 × 10^−6^ and a HSP of >50 bits. Of the clones that contributed to these sequences, there were 13,773 where both end reads were linked to unique pairs of zebrafish proteins [68].

### UCEs.

UCEs identified in the mammalian genomes [39] were searched against the elephant shark, fugu, and zebrafish genomes using BLASTN to identify elements that showed a minimum 100 bp alignment with UCEs.

## Supporting Information

Table S1 InterPro Domains Predicted in the Elephant Shark and Mammals but Absent from Teleost Fishes (51 KB DOC)

Table S2 InterPro Domains Predicted in the Elephant Shark and Teleost Fishes but Absent from Mammals (31 KB DOC)

Table S3 InterPro Domains Predicted in the Elephant Shark That Are Absent from Mammals and Teleost Fishes (35 KB DOC)

Table S4 Human Genes That Have Orthologs in the Genomes of Mouse, Dog, and Elephant Shark, but Not Fugu, *Tetraodon,* or Zebrafish (293 KB DOC)

Table S5 Teleost Genes That Have Orthologs in the Genomes of Zebrafish, Fugu, *Tetraodon,* and Elephant Shark, but Not Human, Mouse, or Dog (157 KB DOC)

### Accession Numbers

This Whole-Genome Shotgun project has been deposited at DNA Databank of Japan/EMBL/GenBank under the project accession AAVX00000000. The version described in this paper is the first version, AAVX01000000. The whole-genome shotgun sequences can also be BLAST-searched on our webpage at http://esharkgenome.imcb.a-star.edu.sg. The repetitive sequences identified have been deposited in GenBank (http://www.ncbi.nlm.nih.gov/Genbank) under the accession numbers DQ524329 to DQ524339.

## Abbreviations

2′-5′OAS: 2′-5′oligoadenylate synthetase 1
ENaC: epithelial Na^+^ channel
*GbX*: *globinX*
HSP: high-scoring segment pair
IgNAR: immunoglobulin new antigen receptor
LINE: long interspersed element
MHC: major histocompatibility complex
My: million years
NAR-TcRV: new antigen receptor–T-cell receptor V domain
RAG: recombination activating gene
RNaseL: ribonuclease L
SINE: short interspersed element
UCE: ultraconserved element
ZP: zona pellucida
