# Supplementary material for: Survey Sequencing and Comparative Analysis of the Elephant Shark (Callorhinchus milii) Genome
Source: PLoS Biol. 2007 Apr 3;5(4):e101. doi: 10.1371/journal.pbio.0050101 (PMC1845163; doi:10.1371/journal.pbio.0050101)
Supplement: Supplementary file 1 [file pbio.0050101.st001.doc]

Table S1. InterPro domains predicted in the elephant shark and mammals but absent from teleost fishes.

| **S/No.** | **Interpro ID** | **Interpro Description** |
| --- | --- | --- |
| 1 | IPR000473 | Ribosomal protein L36 |
| 2 | IPR000779 | Interleukin-2 |
| 3 | IPR001133 | NADH-ubiquinone oxidoreductase, chain 4L |
| 4 | IPR001620 | Dopamine D3 receptor |
| 5 | IPR001966 | Gastrin-releasing peptide receptor |
| 6 | IPR002050 | ENV polyprotein (coat polyprotein) |
| 7 | IPR002093 | BRCA2 repeat |
| 8 | IPR002236 | C-C chemokine receptor, type 1 |
| 9 | IPR002260 | Gap junction alpha-9 protein (Cx36) |
| 10 | IPR002280 | Melatonin-related 1X receptor |
| 11 | IPR002436 | Dopamine neurotransmitter transporter |
| 12 | IPR002494 | Keratin, high sulfur B2 protein |
| 13 | IPR002516 | Glycosyl transferase, family 11 |
| 14 | IPR002955 | Tau protein |
| 15 | IPR003566 | T-cell surface glycoprotein CD5 |
| 16 | IPR004069 | C-C chemokine receptor, type 9 |
| 17 | IPR006117 | 2-5-oligoadenylate synthetase |
| 18 | IPR006695 | CENP-B, N-terminal DNA-binding |
| 19 | IPR007421 | Protein of unknown function DUF467 |
| 20 | IPR007577 | Glycosyltransferase sugar-binding region containing DXD motif |
| 21 | IPR007652 | Alpha 1,4-glycosyltransferase conserved region |
| 22 | IPR008696 | NAF1 |
| 23 | IPR009408 | Formin Homology 1 |
| 24 | IPR010457 | Ig-like C2-type |
| 25 | IPR010857 | Zona-pellucida-binding |
| 26 | IPR011679 | Endoplasmic reticulum ERp29, C-terminal |
