# Supplementary material for: Survey Sequencing and Comparative Analysis of the Elephant Shark (Callorhinchus milii) Genome
Source: PLoS Biol. 2007 Apr 3;5(4):e101. doi: 10.1371/journal.pbio.0050101 (PMC1845163; doi:10.1371/journal.pbio.0050101)
Supplement: Supplementary file 2 [file pbio.0050101.st002.doc]

Table S2. InterPro domains predicted in the elephant shark and teleost fishes but absent from mammals.

| **S/No.** | **Interpro ID** | **Interpro Description** |
| --- | --- | --- |
| 1 | IPR000541 | Protein of unknown function UPF0021 |
| 2 | IPR001910 | Inosine/uridine-preferring nucleoside hydrolase |
| 3 | IPR002081 | Deoxyribodipyrimidine photolyase, class 1 |
| 4 | IPR005560 | Protein of unknown function DUF326 |
| 5 | IPR006031 | XYPPX repeat |
