# Supplementary material for: Survey Sequencing and Comparative Analysis of the Elephant Shark (Callorhinchus milii) Genome
Source: PLoS Biol. 2007 Apr 3;5(4):e101. doi: 10.1371/journal.pbio.0050101 (PMC1845163; doi:10.1371/journal.pbio.0050101)
Supplement: Supplementary file 3 [file pbio.0050101.st003.doc]

Table S3. InterPro domains predicted in the elephant shark that are absent from mammals and teleost fishes.

| **S/No.** | **Interpro ID** | **Interpro Description** |
| --- | --- | --- |
| 1 | IPR000890 | Acetate and butyrate kinase |
| 2 | IPR001155 | NADH:flavin oxidoreductase/NADH oxidase |
| 3 | IPR001764 | Glycoside hydrolase, family 3, N-terminal |
| 4 | IPR001869 | Thiol-activated cytolysin |
| 5 | IPR002772 | Glycoside hydrolase, family 3, C-terminal |
| 6 | IPR005529 | Protein of unknown function DUF321 |
| 7 | IPR007214 | YbaK/prolyl-tRNA synthetase associated region |
| 8 | IPR008118 | Dense granule Gra2 protein |
| 9 | IPR010285 | Eukaryotic protein of unknown function DUF889 |
| 10 | IPR010882 | Acidic phosphoprotein PCEMA1 |
