# Supplementary material for: Survey Sequencing and Comparative Analysis of the Elephant Shark (Callorhinchus milii) Genome
Source: PLoS Biol. 2007 Apr 3;5(4):e101. doi: 10.1371/journal.pbio.0050101 (PMC1845163; doi:10.1371/journal.pbio.0050101)
Supplement: Supplementary file 4 [file pbio.0050101.st004.doc]

Table S4. Human genes that have orthologues in the genomes of mouse, dog and elephant shark, but not fugu, *Tetraodon* or zebrafish. Genes that have no homologs in *Ciona*, fruit fly or the nematode worm (BLASTP with 1e-10 cutoff) are indicated with an asterisk.

|  | Ensembl ID | Ensembl description | GO description | InterPro domain |
| --- | --- | --- | --- | --- |
| 1 | ENSP00000343776* | 14 kDa protein |  | Ig-like |
| 2 | ENSP00000351174* | 18 kDa protein |  |  |
| 3 | ENSP00000258306^©^ | 2-5A-dependent ribonuclease (EC 3.1.26.-) (2-5A-dependent RNase) (Ribonuclease L) (RNase L) (Ribonuclease 4). | hydrolase activity; ATP binding; protein amino acid phosphorylation; RNA binding; endoribonuclease activity, producing 5'-phosphomonoesters; mRNA processing; protein kinase activity | ANK; Prot_kinase; Ribonuc_2-5A |
| 4 | ENSP00000202917^©^ | 2'-5'-oligoadenylate synthetase 1 (EC 2.7.7.-) ((2-5')oligo(A) synthetase 1) (2-5A synthetase 1) (p46/p42 OAS) (E18/E16). | ATP binding; transferase activity; RNA binding; immune response; nucleobase, nucleoside, nucleotide and nucleic acid metabolism; response to virus; nucleotidyltransferase activity |  |
| 5 | ENSP00000328502* | 29 kDa protein |  |  |
| 6 | ENSP00000331782 | 47 kDa protein |  | Cyt_B5; Fa_desat |
| 7 | ENSP00000297628 | 51 kDa protein |  | C2 |
| 8 | ENSP00000350962* | 75 kDa protein |  |  |
| 9 | ENSP00000269701* | A-kinase anchor protein 8 (A-kinase anchor protein 95 kDa) (AKAP 95). | DNA binding; transport; zinc ion binding; mitosis; signal transduction; sugar porter activity; protein kinase A binding | AKAP95 |
| 10 | ENSP00000236709 | Alpha-1,4-N-acetylglucosaminyltransferase (EC 2.4.1.-) (Alpha4GnT). | carbohydrate metabolism; transferase activity, transferring glycosyl groups; galactosyltransferase activity; protein amino acid O-linked glycosylation; acetylglucosaminyltransferase activity | Gly_transf_sug; Gb3_synth |
| 11 | ENSP00000264432 | amiloride-sensitive cation channel 5, intestinal | sodium ion transport; ion transport; ion channel activity; sodium channel activity | Na+channel_ASC |
| 12 | ENSP00000302874 | Amiloride-sensitive sodium channel beta-subunit (Epithelial Na+ channel beta subunit) (Beta ENaC) (Nonvoltage-gated sodium channel 1 beta subunit) (SCNEB) (Beta NaCH) (ENaCB). | sodium ion transport; ion transport; ion channel activity; excretion; amiloride-sensitive sodium channel activity | Na+channel_ASC |
| 13 | ENSP00000300061 | Amiloride-sensitive sodium channel gamma-subunit (Epithelial Na+ channel gamma subunit) (Gamma ENaC) (Nonvoltage-gated sodium channel 1 gamma subunit) (SCNEG) (Gamma NaCH). | sodium ion transport; ion transport; ion channel activity; excretion; amiloride-sensitive sodium channel activity | Na+channel_ASC |
| 14 | ENSP00000284142* | Ankyrin repeat and SOCS box protein 17 (ASB-17). | intracellular signaling cascade | SOCS_C |
| 15 | ENSP00000347538 | B/K protein | transporter activity; transport | C2 |
| 16 | ENSP00000294379* | Barttin. |  |  |
| 17 | ENSP00000320509* | B-cell scaffold protein with ankyrin repeats 1 |  |  |
| 18 | ENSP00000281445 | BH3-only member B protein |  | C2 |
| 19 | ENSP00000337181 | Boule-like protein. | RNA binding; spermatogenesis; regulation of translation | RNA_rec_mot |
| 20 | ENSP00000346073* | CGG triplet repeat binding protein 1 | double-stranded DNA binding |  |
| 21 | ENSP00000308214* | CMT duplicated region transcript 1. |  |  |
| 22 | ENSP00000259938* | Colipase precursor. | lipid catabolism; digestion; enzyme activator activity | Colipase |
| 23 | ENSP00000272995 | COP9 signalosome complex subunit 7b (Signalosome subunit 7b) (SGN7b) (JAB1-containing signalosome subunit 7b). |  | PCI |
| 24 | ENSP00000296161 | Deltex 3-like protein (B-lymphoma- and BAL-associated protein) (Rhysin-2) (Rhysin2). | ubiquitin-protein ligase activity; zinc ion binding; protein ubiquitination |  |
| 25 | ENSP00000335085 | Doublecortin domain-containing protein 1. | intracellular signaling cascade |  |
| 26 | ENSP00000261735 | Endoplasmic reticulum protein ERp29 precursor (ERp31) (ERp28). | protein folding; intracellular protein transport; protein disulfide isomerase activity | ERp29 |
| 27 | ENSP00000320498* | erythroblast membrane-associated protein |  | SPRY_receptor |
| 28 | ENSP00000321386 | F-box only protein 39. | ubiquitin cycle | F-box |
| 29 | ENSP00000284224 | GalNAc-4-sulfotransferase 2 | transferase activity; central nervous system development; N-acetylgalactosamine 4-O-sulfotransferase activity; glycosaminoglycan metabolism; proteoglycan biosynthesis; hormone biosynthesis | Sulfotransfer2 |
| 30 | ENSP00000295256 | Glutathione-requiring prostaglandin D synthase (EC 5.3.99.2) (Glutathione-dependent PGD synthetase) (Prostaglandin-H2 D-isomerase) (Hematopoietic prostaglandin D synthase) (H-PGDS). | signal transduction; isomerase activity; cell-cell signaling; locomotory behavior; prostaglandin-D synthase activity; prostaglandin biosynthesis | GST_Nterm; GST_Cterm |
| 31 | ENSP00000343950 | Heat shock transcription factor 1 (Fragment). | regulation of transcription, DNA-dependent; protein folding; transcription factor activity; response to unfolded protein | HSF_DNA_bind; Vert_HS_TF |
| 32 | ENSP00000302476 | Heat-shock protein beta-2 (HspB2) (DMPK-binding protein) (MKBP). | protein binding; protein folding; response to unfolded protein; enzyme activator activity | Hsp20 |
| 33 | ENSP00000307954* | HGS_RE408 |  | DUF1180 |
| 34 | ENSP00000242159 | Homeobox protein Hox-A7 (Hox-1A) (Hox 1.1). | development; regulation of transcription, DNA-dependent; transcription factor activity | Homeobox |
| 35 | ENSP00000315949 | Homeobox protein Hox-D8 (Hox-4E) (Hox-5.4). | development; regulation of transcription, DNA-dependent; transcription factor activity; determination of anterior/posterior axis, embryo | Homeobox |
| 36 | ENSP00000283429* | HSCARG protein |  |  |
| 37 | ENSP00000259676 | insulin-like growth factor binding protein-like 1 | regulation of cell growth; insulin-like growth factor binding | Prot_inh_Kazal; Prot_Inh_Kazal_2; Ig-like |
| 38 | ENSP00000322196* | ITBA1 protein (CXorf12 protein) (DXS9878E). | molecular function unknown; biological process unknown |  |
| 39 | ENSP00000343636* | IVFI9356 | receptor activity | GDNF_receptor |
| 40 | ENSP00000306929 | LYST-interacting protein LIP8 |  |  |
| 41 | ENSP00000273432 | mediator of RNA polymerase II transcription, subunit 12 homolog (yeast)-like | receptor activity |  |
| 42 | ENSP00000325280 | Melanoma-associated antigen D1 (MAGE-D1 antigen) (Neurotrophin receptor-interacting MAGE homolog) (PRO2292) (PP2250). |  | MAGE |
| 43 | ENSP00000295896* | MGC17310 protein. |  |  |
| 44 | ENSP00000341174* | Novel protein (Fragment). |  |  |
| 45 | ENSP00000310848* | Novel protein (Fragment). |  |  |
| 46 | ENSP00000253255 | Polycystic kidney disease and receptor for egg jelly related protein precursor (PKD and REJ homolog). | cation channel activity; calcium ion binding; cation transport; neuropeptide signaling pathway | PKD/REJ-like; Lipoxygenase_LH2; Ion_trans |
| 47 | ENSP00000326701* | Potassium voltage-gated channel subfamily E member 1-like protein (AMME syndrome candidate gene 2 protein) (AMMECR2 protein). | sensory perception of sound; regulation of heart contraction |  |
| 48 | ENSP00000246074 | precursor. |  | LRR; FN_III |
| 49 | ENSP00000252307 | PREDICTED: chromosome 6 open reading frame 70 |  |  |
| 50 | ENSP00000315769* | PREDICTED: FLJ34306 protein |  |  |
| 51 | ENSP00000182096* | PREDICTED: hypothetical protein DKFZp667G2110 | sugar binding | Crystallin; Ricin_B_lectin |
| 52 | ENSP00000327681* | PREDICTED: hypothetical protein MGC35030 |  |  |
| 53 | ENSP00000323821* | PREDICTED: hypothetical protein XP_370638 |  |  |
| 54 | ENSP00000300643* | PREDICTED: hypothetical protein XP_371053 |  |  |
| 55 | ENSP00000334072* | PREDICTED: hypothetical protein XP_371680 |  |  |
| 56 | ENSP00000345619* | PREDICTED: hypothetical protein XP_374437 |  |  |
| 57 | ENSP00000331888 | PREDICTED: hypothetical protein XP_376372 |  |  |
| 58 | ENSP00000340752* | PREDICTED: hypothetical protein XP_499602 |  |  |
| 59 | ENSP00000301807* | PREDICTED: KIAA0342 gene product | DNA binding |  |
| 60 | ENSP00000341803 | PREDICTED: similar to hypothetical protein 4833401D15 |  | Tctex |
| 61 | ENSP00000305702* | PREDICTED: similar to hypothetical protein 4922504H04 | voltage-gated potassium channel activity; potassium ion transport | K_tetra |
| 62 | ENSP00000284630 | PREDICTED: similar to hypothetical protein FLJ20035 | ATP binding; helicase activity | ResIII; DEAD/DEAH_N |
| 63 | ENSP00000346943* | PREDICTED: similar to LRRGT00163 |  | DUF467 |
| 64 | ENSP00000328159 | PREDICTED: similar to RIKEN cDNA 1700007I06 | calcium ion binding | EF-hand |
| 65 | ENSP00000299866 | PREDICTED: similar to RIKEN cDNA 1810036I24 |  | DUF846 |
| 66 | ENSP00000344720* | PREDICTED: similar to RIKEN cDNA 2310010M20 |  |  |
| 67 | ENSP00000317088 | prematurely terminated mRNA decay factor-like | zinc ion binding | ZF-GRF |
| 68 | ENSP00000334654* | pre-mRNA splicing factor-like |  | WD40 |
| 69 | ENSP00000351088 | proapoptotic caspase adaptor protein |  |  |
| 70 | ENSP00000325807* | Probable G-protein coupled receptor 150. | rhodopsin-like receptor activity; G-protein coupled receptor protein signaling pathway | GPCR_Rhodpsn |
| 71 | ENSP00000351379* | Profilin-3 (Profilin III). | actin binding; actin cytoskeleton organization and biogenesis; cytoskeleton organization and biogenesis | Profilin |
| 72 | ENSP00000296506* | Scrapie-responsive protein 1 precursor (ScRG-1) (UNQ390/PRO725). | nervous system development |  |
| 73 | ENSP00000242059 | Secernin 1. | molecular function unknown; proteolysis; exocytosis; dipeptidase activity | Peptidase_U34 |
| 74 | ENSP00000264852 | SID1 transmembrane family, member 1 |  |  |
| 75 | ENSP00000345468* | similar to RIKEN A930001M12 |  |  |
| 76 | ENSP00000238138 | SMP3 mannosyltransferase | RNA binding; transferase activity, transferring glycosyl groups; ribonuclease H activity | Alg9_trans |
| 77 | ENSP00000275764* | STRA8 |  | HLH_basic |
| 78 | ENSP00000300692* | T-cell surface glycoprotein CD3 delta chain precursor (T-cell receptor T3 delta chain). | protein binding; protein heterodimerization activity; protein complex assembly; transmembrane receptor activity; cell surface receptor linked signal transduction; T cell activation; positive thymic T cell selection | ITAM |
| 79 | ENSP00000326652* | testes development-related NYD-SP18 |  |  |
| 80 | ENSP00000240361 | testis expressed sequence 14 isoform b | ATP binding; protein amino acid phosphorylation; protein kinase activity | ANK; Prot_kinase |
| 81 | ENSP00000310796* | THAP domain protein 2. | DNA binding | THAP_DM3 |
| 82 | ENSP00000292894* | THAP domain protein 8. | DNA binding | THAP_DM3 |
| 83 | ENSP00000246024 | Thioredoxin-like | electron transport; electron transporter activity | Thiored |
| 84 | ENSP00000308354* | tigger transposable element derived 3 | DNA binding | CENP-B_N; CENP-B |
| 85 | ENSP00000296736 | tigger transposable element derived 6 | DNA binding; regulation of transcription | CENP-B_N; CENP-B |
| 86 | ENSP00000248244* | TIR domain containing adaptor inducing interferon-beta | inflammatory response; transmembrane receptor activity; signal transducer activity; positive regulation of I-kappaB kinase/NF-kappaB cascade |  |
| 87 | ENSP00000333650* | toll-like receptor adaptor molecule 2 | intracellular protein transport; protein carrier activity; signal transducer activity; inflammatory response; transmembrane receptor activity; positive regulation of I-kappaB kinase/NF-kappaB cascade | Emp24_gp25L_p24 |
| 88 | ENSP00000265007 | Transcription factor SOX-30. | DNA binding; spermatogenesis; RNA polymerase II transcription factor activity; regulation of transcription from RNA polymerase II promoter | HMG_12_box |
| 89 | ENSP00000286349* | Tripartite motif protein 42. | ubiquitin-protein ligase activity; zinc ion binding; protein ubiquitination | Znf_ring; Znf_Bbox; FN_III |
| 90 | ENSP00000305161 | Tripartite motif protein 56 (RING finger protein 109). | ubiquitin-protein ligase activity; zinc ion binding; protein ubiquitination | Znf_ring; Znf_Bbox |
| 91 | ENSP00000216211* | Uroplakin-3A precursor (Uroplakin III) (UPIII). |  |  |
| 92 | ENSP00000269499* | Zinc finger CCHC domain containing protein 2 (Fragment). | nucleic acid binding | Znf_CCHC |
| 93 | ENSP00000046087* | Zona pellucida binding protein 1 precursor (Sp38). |  | Sp38 |
| 94 | ENSP00000278853* | Zona pellucida sperm-binding protein 1 precursor (Zona pellucida glycoprotein 1) (Zp-1). | fertilization (sensu Metazoa) | Endoglin/CD105 |
| 95 | ENSP00000301714* |  |  |  |
| 96 | ENSP00000340960 |  |  | WD40 |
| 97 | ENSP00000268492* |  | sugar binding | Lectin_C |
| 98 | ENSP00000315226 |  |  |  |
| 99 | ENSP00000344013 |  |  | WD40 |
| 100 | ENSP00000257766 |  |  |  |
| 101 | ENSP00000342799* |  |  |  |
| 102 | ENSP00000244062 |  | protein modification | ANK |
| 103 | ENSP00000311665 |  |  | WD40 |
| 104 | ENSP00000310951* |  |  |  |
| 105 | ENSP00000320911 |  | binding |  |
| 106 | ENSP00000314914* |  |  |  |
| 107 | ENSP00000306516* |  |  |  |
| 108 | ENSP00000333392 |  |  | LRR |
| 109 | ENSP00000331295* |  | catalytic activity |  |
| 110 | ENSP00000339589 |  |  |  |
| 111 | ENSP00000288065* |  | binding |  |
| 112 | ENSP00000310622* |  |  |  |
| 113 | ENSP00000301896* |  |  |  |
| 114 | ENSP00000324241* |  |  |  |
| 115 | ENSP00000296600 |  | nucleic acid binding; zinc ion binding | Znf_C2H2 |
| 116 | ENSP00000345555 |  |  | Enolase |
| 117 | ENSP00000321788* |  |  |  |
| 118 | ENSP00000298466* |  |  | HLH_basic |
| 119 | ENSP00000311314* |  |  |  |
| 120 | ENSP00000279026* |  |  |  |
| 121 | ENSP00000287709 |  |  |  |
| 122 | ENSP00000343496* |  |  |  |
| 123 | ENSP00000293440 |  |  |  |
| 124 | ENSP00000325012 |  |  |  |
| 125 | ENSP00000332875 |  |  |  |
| 126 | ENSP00000226432 |  |  |  |
| 127 | ENSP00000230301 |  |  |  |
| 128 | ENSP00000335612 |  | calcium ion binding | EF-hand |
| 129 | ENSP00000297887 |  |  |  |
| 130 | ENSP00000287899* |  | oxidoreductase activity; electron transport | Oxred_FAD/NAD(P); CUB |
| 131 | ENSP00000318825 |  |  |  |
| 132 | ENSP00000342719* |  |  |  |
| 133 | ENSP00000352397* |  |  |  |
| 134 | ENSP00000316547* |  |  | cNMP_binding |
| 135 | ENSP00000341700 |  | intracellular signaling cascade | DCX |
| 136 | ENSP00000271416 |  | DNA binding |  |
| 137 | ENSP00000245671* |  |  |  |
| 138 | ENSP00000310513* |  |  |  |
| 139 | ENSP00000304401* |  |  | DUF467 |
| 140 | ENSP00000314477* |  |  |  |
| 141 | ENSP00000282881 |  |  | IF_tail_C |
| 142 | ENSP00000324920* |  |  |  |
| 143 | ENSP00000299191* |  |  |  |
| 144 | ENSP00000313280 |  |  |  |
| 145 | ENSP00000284694* |  |  |  |
| 146 | ENSP00000327812* |  |  |  |
| 147 | ENSP00000321179* |  |  |  |
| 148 | ENSP00000158009* |  |  | FN_III |
| 149 | ENSP00000287773* |  |  |  |
| 150 | ENSP00000294607* |  | calcium ion binding |  |
| 151 | ENSP00000341451* |  |  |  |
| 152 | ENSP00000332663* |  |  |  |
| 153 | ENSP00000323355* |  |  |  |
| 154 | ENSP00000244073 |  |  |  |

^©^although some genes in *Ciona* share homology with these genes because of shared domains, they are not the orthologs of these genes.
