# Supplementary material for: Survey Sequencing and Comparative Analysis of the Elephant Shark (Callorhinchus milii) Genome
Source: PLoS Biol. 2007 Apr 3;5(4):e101. doi: 10.1371/journal.pbio.0050101 (PMC1845163; doi:10.1371/journal.pbio.0050101)
Supplement: Supplementary file 5 [file pbio.0050101.st005.doc]

Table S5. Teleost genes that have orthologues in the genomes of zebrafish (listed below), fugu, *Tetraodon* and elephant shark, but not human, mouse or dog. Genes that have no homologs in *Ciona*, fruit fly or the nematode worm (BLASTP with 1e-10 cutoff) are indicated with an asterisk.

|  | Ensembl ID | Ensembl Description | GO description | Interpro |
| --- | --- | --- | --- | --- |
| 1 | ENSDARP00000046413 | FYN oncogene related to SRC, FGR, YES (fyn), mRNA |  | SH2 motif; SH3; Variant SH3 |
| 2 | ENSDARP00000040367 | homeo box HB9 like b | regulation of transcription, DNA-dependent; DNA binding; transcription factor activity | Homeobox |
| 3 | ENSDARP00000001191 | Homeobox protein Hox-B1b (Hox-A1). | regulation of transcription, DNA-dependent; DNA binding; transcription factor activity; development; transcription; hindbrain development | Helix-turn-helix motif, lambda-like repressor; Homeobox |
| 4 | ENSDARP00000032215 | Hypothetical protein (Fragment). |  | Peptidase M17, leucyl aminopeptidase, C-terminal; IMP dehydrogenase/GMP reductase |
| 5 | ENSDARP00000033960 | Hypothetical protein (Fragment). |  |  |
| 6 | ENSDARP00000049967 | Hypothetical protein (Fragment). |  | Bipartite nuclear localization signal |
| 7 | ENSDARP00000048321 | Hypothetical protein (Fragment). |  | Zn-finger, B-box; Zn-finger, RING |
| 8 | ENSDARP00000032457* | hypothetical protein LOC326023 | molecular function unknown; biological process unknown | IMP dehydrogenase/GMP reductase |
| 9 | ENSDARP00000000436* | hypothetical protein LOC368688 | transport; oxidoreductase activity; electron transport | IMP dehydrogenase/GMP reductase; Bipartite nuclear localization signal; Protein of unknown function DUF455 |
| 10 | ENSDARP00000036049* | hypothetical protein LOC393502 | biological process unknown; calcium ion binding | Aspartic acid and asparagine hydroxylation site; CUB; EGF-like calcium-binding |
| 11 | ENSDARP00000063952 | hypothetical protein LOC393525 | metabolism; oxidoreductase activity | Short-chain dehydrogenase/reductase SDR; Glucose/ribitol dehydrogenase |
| 12 | ENSDARP00000011222 | hypothetical protein LOC405817 | catalytic activity; hydrolase activity; protein amino acid dephosphorylation; phosphoprotein phosphatase activity; magnesium ion binding; protein serine/threonine phosphatase activity; manganese ion binding | Protein phosphatase 2C; Protein phosphatase 2C-like |
| 13 | ENSDARP00000010433 | hypothetical protein LOC406725 | molecular function unknown; biological process unknown | GNS1/SUR4 membrane protein |
| 14 | ENSDARP00000020993 | hypothetical protein LOC436638 | chromatin modification; chromatin binding; chromatin assembly or disassembly | IMP dehydrogenase/GMP reductase; Bipartite nuclear localization signal; MOZ/SAS-like protein |
| 15 | ENSDARP00000049035 | hypothetical protein LOC436885 | transport; oxidoreductase activity; electron transport | Rieske [2Fe-2S] region |
| 16 | ENSDARP00000009016 | hypothetical protein LOC436996 | ubiquitin-protein ligase activity; protein ubiquitination; zinc ion binding | Zn-finger, B-box; Zn-finger, RING |
| 17 | ENSDARP00000055847 | hypothetical protein LOC494056 | hydrolase activity; biological process unknown; hydrolase activity, acting on carbon-nitrogen (but not peptide) bonds, in cyclic amides | IMP dehydrogenase/GMP reductase; TatD-related deoxyribonuclease; Dihydroorotase; Amidohydrolase |
| 18 | ENSDARP00000063079 | hypothetical protein LOC541424 | molecular function unknown; biological process unknown | CD9/CD37/CD63 antigen |
| 19 | ENSDARP00000017469 | hypothetical protein LOC550326 | molecular function unknown; biological process unknown | Peptidase, cysteine peptidase active site; Peptidase C1A, papain; IMP dehydrogenase/GMP reductase; Peptidase C1, propeptide |
| 20 | ENSDARP00000017026 | hypothetical protein LOC550589 | molecular function unknown; biological process unknown | Bipartite nuclear localization signal; Peptidase M10A and M12B, matrixin and adamalysin; Metridin-like ShK toxin; Peptidase M, neutral zinc metallopeptidases, zinc-binding site |
| 21 | ENSDARP00000016820* | hypothetical protein LOC550608 | molecular function unknown; biological process unknown | Apolipoprotein A1/A4/E |
| 22 | ENSDARP00000058323* | hypothetical protein LOC550609 | molecular function unknown; biological process unknown |  |
| 23 | ENSDARP00000028675 | hypothetical protein zgc:85777 | oxidoreductase activity; electron transport; acyl-CoA dehydrogenase activity | IMP dehydrogenase/GMP reductase; Acyl-CoA dehydrogenase; Acyl-CoA dehydrogenase, C-terminal; Acyl-CoA dehydrogenase, central region; Acyl-CoA dehydrogenase, N-terminal |
| 24 | ENSDARP00000061735 | LOC402865 protein (Fragment). |  | IMP dehydrogenase/GMP reductase; Inosine/uridine-preferring nucleoside hydrolase |
| 25 | ENSDARP00000063018 | muscle-specific beta 1 integrin binding protein 2 | ATP binding; transferase activity; kinase activity; uridine kinase activity; pyridine nucleotide biosynthesis; integrin-mediated signaling pathway | Uridine kinase; ATP/GTP-binding site motif A (P-loop) |
| 26 | ENSDARP00000035057 | neurofibromin 2 (bilateral acoustic neuroma) | cytoskeletal protein binding | Band 4.1; Tropomyosin; Ezrin/radixin/moesin ERM; Ezrin/radixin/moesin, C-terminal |
| 27 | ENSDARP00000044545* | Novel protein (Fragment). |  |  |
| 28 | ENSDARP00000063180 | Novel protein (Fragment). |  |  |
| 29 | ENSDARP00000003210 | Novel protein containing a beta-ketoacyl synthase, N-terminal domain (Fragment). |  | Beta-ketoacyl synthase; IMP dehydrogenase/GMP reductase; Acyl transferase region; Short-chain dehydrogenase/reductase SDR; Phosphopantetheine-binding |
| 30 | ENSDARP00000043495 | Novel protein containing a homocysteine S-methyltransferase domain (Fragment). |  | IMP dehydrogenase/GMP reductase; Homocysteine S-methyltransferase |
| 31 | ENSDARP00000063702 | Novel protein containing a phosphotyrosine interaction domain (PTB/PID) (Fragment). |  | Bipartite nuclear localization signal; Phosphotyrosine interaction region |
| 32 | ENSDARP00000029516 | Novel protein similar to vertebrate ancient long opsin (Valop) (Fragment). | receptor activity; rhodopsin-like receptor activity; G-protein coupled receptor protein signaling pathway; G-protein coupled receptor activity; amino acid metabolism; visual perception | Rhodopsin-like GPCR superfamily; Opsin; Asparaginase/glutaminase |
| 33 | ENSDARP00000061755 | Novel protein similar to vertebrate phosphohistidine phosphatase 1 (PHPT1). |  | Janus/Ocnus |
| 34 | ENSDARP00000052666 | Novel protein similar to vertebrate RAS-like, estrogen-regulated, growth-inhibitor (RERG). |  | ATP/GTP-binding site motif A (P-loop); Ras GTPase |
| 35 | ENSDARP00000064500* | Novel protein. |  | Proline-rich region |
| 36 | ENSDARP00000044779* | Novel zona pellucida-like domain containg protein. |  | IMP dehydrogenase/GMP reductase; Endoglin/CD105 antigen |
| 37 | ENSDARP00000061326 | Rh50-like protein |  | IMP dehydrogenase/GMP reductase; Blood group Rhesus C/E and D polypeptide; Rh-like protein/ammonium transporter |
| 38 | ENSDARP00000024068 | similar to WAS protein family, member 2 | molecular function unknown; biological process unknown | Bipartite nuclear localization signal |
| 39 | ENSDARP00000009745 | southpaw | growth factor activity; determination of left/right symmetry | Transforming growth factor beta; Inhibin, alpha subunit |
| 40 | ENSDARP00000027246 | Unknown (protein for MGC:63758) | transport; transporter activity; tetracycline transport; tetracycline:hydrogen antiporter activity | IMP dehydrogenase/GMP reductase; Tetracycline resistance protein; Sugar transporter superfamily; Major facilitator superfamily MFS_1 |
| 41 | ENSDARP00000050237 | vitellogenin 1 | lipid transport; lipid transporter activity | IMP dehydrogenase/GMP reductase; Lipid transport protein, N-terminal |
| 42 | ENSDARP00000056380 | Wu:fi20c07 protein (Fragment). |  | Pentaxin |
| 43 | ENSDARP00000045749* | x globin | transport; binding; oxygen transport; oxygen transporter activity; oxygen binding | Globin; Leghaemoglobin |
| 44 | ENSDARP00000040854 | zgc:101777 | molecular function unknown; biological process unknown | Cdc15/Fes/CIP4; IMP dehydrogenase/GMP reductase |
| 45 | ENSDARP00000047112 | zgc:77651 (zgc:77651), mRNA |  | Esterase/lipase/thioesterase; IMP dehydrogenase/GMP reductase; Acyl-CoA thioester hydrolase/bile acid-CoA amino acid N-acetyltransferase |
| 46 | ENSDARP00000065954 | zgc:92107 | molecular function unknown; biological process unknown | Unconventional myosin/plant kinesin-like protein/non-motor protein conserved region MyTH4; IMP dehydrogenase/GMP reductase; Bipartite nuclear localization signal |
| 47 | ENSDARP00000056814 | zgc:92276 | intracellular protein transport; protein transport; GTP binding; small GTPase mediated signal transduction | ATP/GTP-binding site motif A (P-loop); Ras GTPase |
| 48 | ENSDARP00000055462* |  |  | IMP dehydrogenase/GMP reductase |
| 49 | ENSDARP00000064058 |  |  | SAM (and some other nucleotide) binding motif; Generic methyltransferase; Cyclopropane-fatty-acyl-phospholipid synthase; UbiE/COQ5 methyltransferase |
| 50 | ENSDARP00000010368* |  |  | Oxidoreductase, N-terminal; IMP dehydrogenase/GMP reductase |
| 51 | ENSDARP00000058698* |  |  | Coagulation factor 5/8 type, C-terminal |
| 52 | ENSDARP00000056739 |  |  | Malate synthase |
| 53 | ENSDARP00000027041 |  |  | Adenylate cyclase; Uncharacterised conserved protein CYTH, CHAD, and HD-like; Uncharacterised conserved protein CYTH and CHAD regions, YgiF-related |
| 54 | ENSDARP00000063736 |  |  | SOUL heme-binding protein |
| 55 | ENSDARP00000045328 |  |  | Membrane-associated proteins in eicosanoid and glutathione metabolism (MAPEG); TonB box, N-terminal |
| 56 | ENSDARP00000059674 |  |  | IMP dehydrogenase/GMP reductase; Bipartite nuclear localization signal; Photosystem I assembly BtpA |
| 57 | ENSDARP00000062421 |  |  |  |
| 58 | ENSDARP00000059072 |  |  | SUA5/yciO/yrdC, N-terminal |
| 59 | ENSDARP00000057518 |  |  | Strictosidine synthase |
| 60 | ENSDARP00000057263 |  |  | SOUL heme-binding protein |
| 61 | ENSDARP00000062166* |  |  | Rhodopsin-like GPCR superfamily |
| 62 | ENSDARP00000067437 |  |  | Calponin-like actin-binding |
| 63 | ENSDARP00000063777 |  |  | Ca2+/Na+ channel, pore region; ATP/GTP-binding site motif A (P-loop); Cation (not K+) channel, TM region; Cation channel, non-ligand gated; Ion transport protein |
| 64 | ENSDARP00000042545* |  |  | Prominin |
| 65 | ENSDARP00000027338 |  |  | CD9/CD37/CD63 antigen |
| 66 | ENSDARP00000060659 |  |  | C-type lectin |
| 67 | ENSDARP00000059554 |  |  | Immunoglobulin-like |
| 68 | ENSDARP00000048641 |  |  | Bipartite nuclear localization signal; Troponin |
| 69 | ENSDARP00000025855 |  |  | Protein of unknown function DUF803 |
| 70 | ENSDARP00000059432 |  |  | RA; IMP dehydrogenase/GMP reductase; PDZ/DHR/GLGF; Dilute |
| 71 | ENSDARP00000062901 |  |  | Basic helix-loop-helix dimerisation region bHLH |
| 72 | ENSDARP00000062035* |  |  |  |
| 73 | ENSDARP00000051886 |  |  | Peptidase T2, asparaginase 2; IMP dehydrogenase/GMP reductase |
| 74 | ENSDARP00000009591 |  |  | Protein kinase; Protein kinase, C-terminal; Phox-like; Serine/threonine protein kinase, active site |
| 75 | ENSDARP00000015980 |  |  | RabGAP/TBC |
| 76 | ENSDARP00000006380 |  |  | IMP dehydrogenase/GMP reductase; TPR repeat; GoLoco; Tetratricopeptide TPR_4 |
| 77 | ENSDARP00000058464* |  |  | FGF binding 1 |
| 78 | ENSDARP00000055616 |  |  | Histone deacetylase superfamily; IMP dehydrogenase/GMP reductase |
| 79 | ENSDARP00000064105 |  |  | ABC-2; ATP/GTP-binding site motif A (P-loop); ABC transporter related |
| 80 | ENSDARP00000047377* |  |  | Somatostatin/Cortistatin |
| 81 | ENSDARP00000026409 |  |  |  |
| 82 | ENSDARP00000055637 |  |  | Leucine-rich repeat |
| 83 | ENSDARP00000014590 |  |  | Connexins |
| 84 | ENSDARP00000052683 |  |  | Sterile alpha motif SAM |
| 85 | ENSDARP00000058387 |  |  | CUB |
| 86 | ENSDARP00000058751 |  |  | Peptidase M20; Peptidase dimerisation |
| 87 | ENSDARP00000065099 |  |  | IMP dehydrogenase/GMP reductase; DEAD/DEAH box helicase; Helicase, C-terminal; DEAD/DEAH box helicase, N-terminal |
| 88 | ENSDARP00000062543 |  |  | Protein of unknown function RIO1; IMP dehydrogenase/GMP reductase; Bipartite nuclear localization signal; Tyrosine protein kinase, active site |
| 89 | ENSDARP00000035291 |  |  | PAS; IMP dehydrogenase/GMP reductase |
| 90 | ENSDARP00000040020 |  |  | Acyltransferase ChoActase/COT/CPT; IMP dehydrogenase/GMP reductase |
| 91 | ENSDARP00000030910 |  |  | RA; Proline-rich region; Pleckstrin-like |
| 92 | ENSDARP00000031910 |  |  | Rhodopsin-like GPCR superfamily; Opsin; Peropsin; 2Fe-2S ferredoxin, iron-sulfur binding site |
| 93 | ENSDARP00000028733 |  |  | Cdc20/Fizzy; IMP dehydrogenase/GMP reductase; WD-40 repeat |
| 94 | ENSDARP00000016731 |  |  | RhoGAP; IMP dehydrogenase/GMP reductase; WW/Rsp5/WWP; SH3; Pleckstrin-like |
| 95 | ENSDARP00000037907 |  |  | RNA-directed DNA polymerase (Reverse transcriptase); IMP dehydrogenase/GMP reductase; Endonuclease/exonuclease/phosphatase |
| 96 | ENSDARP00000051242* |  |  | Bipartite nuclear localization signal; PMP-22/EMP/MP20 and claudin; Claudin tight junction protein |
| 97 | ENSDARP00000048809 |  |  | Ubiquitin-activating enzyme, E1; Ubiquitin-activating enzyme repeat; UBA/THIF-type NAD/FAD binding fold; IMP dehydrogenase/GMP reductase |
| 98 | ENSDARP00000056606 |  |  | Proline-rich region; Collagen triple helix repeat |
| 99 | ENSDARP00000053010* |  |  | Leucine-rich repeat; ATP/GTP-binding site motif A (P-loop) |
| 100 | ENSDARP00000058244* |  |  | Caspase Recruitment |
| 101 | ENSDARP00000045336 |  |  | IMP dehydrogenase/GMP reductase; Exocyst complex component Sec6 |
| 102 | ENSDARP00000010904 |  |  | Helix-turn-helix motif, lambda-like repressor; Homeobox |
| 103 | ENSDARP00000061890 |  |  | IMP dehydrogenase/GMP reductase; Bipartite nuclear localization signal; Actin-binding, actinin-type; Spectrin/pleckstrin-like; Calponin-like actin-binding; Pleckstrin-like; Spectrin repeat |
| 104 | ENSDARP00000039074 |  |  | Semaphorin/CD100 antigen; Plexin; Immunoglobulin-like |
| 105 | ENSDARP00000041430 |  |  | IMP dehydrogenase/GMP reductase; Short-chain dehydrogenase/reductase SDR; Glucose/ribitol dehydrogenase; 2,3-dihydro-2,3-dihydroxybenzoate dehydrogenase |
| 106 | ENSDARP00000065338 |  |  |  |
| 107 | ENSDARP00000055807 |  |  | GCN5-related N-acetyltransferase |
